# Unraveling the multifaceted roles of extracellular vesicles in bladder cancer: diagnostic insights and therapeutic opportunities

**DOI:** 10.3389/fonc.2025.1554819

**Published:** 2025-05-06

**Authors:** Kaiqi Huang, Chen Yang, Yanfang Xu, Yujia Wang

**Affiliations:** ^1^ Department of Nephrology, Blood Purification Research Center, The First Affiliated Hospital, Fujian Medical University, Fuzhou, China; ^2^ Department of Nephrology, National Regional Medical Center, Binhai Campus of the First Affiliated Hospital, Fujian Medical University, Fuzhou, China; ^3^ Fujian Clinical Research Center for Metabolic Chronic Kidney Disease, The First Affiliated Hospital, Fujian Medical University, Fuzhou, China; ^4^ Department of Urology, Huashan Hospital, Fudan University, Shanghai, China

**Keywords:** bladder cancer, extracellular vesicle, tumor microenvironment, biomarker, drug delivery

## Abstract

Bladder cancer, predominantly urothelial carcinoma, is a global health issue with increasing incidences and mortality. It poses significant diagnostic and therapeutic challenges due to its molecular heterogeneity and the limitations of current detection methods. Extracellular vesicles (EVs), including exosomes, play a crucial role in intercellular communication and have emerged as potential biomarkers and therapeutic agents in bladder cancer. This review focuses on the multifaceted roles of EVs in bladder cancer biology, their potential as diagnostic biomarkers, and their use in therapeutic strategies. We discuss how EVs reflect molecular subtypes of bladder cancer, participate in metabolic reprogramming and angiogenesis, and modulate cellular behavior. The review also highlights the advances in proteomic analysis of urinary and tissue-exudative EVs, identifying specific proteins and RNAs that could serve as non-invasive diagnostic markers. Furthermore, we explore the innovative use of EVs as natural nanocarriers for drug delivery in bladder cancer treatment, demonstrating their potential to enhance the efficacy of chemotherapy and selectively target cancer cells. The integration of EV-based diagnostics with traditional methods could lead to more personalized and effective bladder cancer management, emphasizing the need for further research and clinical validation.

## 1 Introduction

Bladder cancer, predominantly characterized by urothelial carcinoma, stands as a significant global health concern due to its increasing incidence and mortality rates. The Global Cancer Statistics 2018 report ranked it as the 12th most frequently diagnosed cancer globally, with an annual estimate of 549,000 new cases and nearly 200,000 fatalities (1). In 2024, an estimated 83,190 new cases of urinary bladder cancer and 16,840 deaths were projected to occur in the United States (2). The disease poses a considerable burden on healthcare systems, not only due to its prevalence but also because of the complexity in diagnosis and treatment, which often involves a multidisciplinary approach (3).

Bladder cancer, divided into non-muscle-invasive (NMIBC) and muscle-invasive (MIBC), presents distinct diagnostic and therapeutic challenges. MIBC, which accounts for 25% of cases at diagnosis, carries a worse prognosis due to its invasiveness and metastatic potential (4). Despite progress, managing bladder cancer remains challenging, with bacillus Calmette-Guérin (BCG) often failing in NMIBC and MIBC necessitating aggressive treatments that affect quality of life (5, 6). The diagnosis of bladder cancer typically involves a combination of cystoscopy, urinary cytology, and imaging studies, which aid in staging and assessing the extent of the disease. However, the sensitivity and specificity of these diagnostic modalities are not infallible, leading to a continuous search for more accurate biomarkers and diagnostic tools (7). The heterogeneity of bladder cancer and the variability in molecular characterization of tumors underscore the complexity of the disease, demanding a tailored approach to therapy that takes into account the specific molecular signatures and histological subtypes of individual tumors (8). This complexity, coupled with the need for improved early detection and personalized treatment strategies, presents the current challenges in the field of bladder cancer management.

The tumor microenvironment (TME) is a complex ecosystem composed of cancer cells, immune cells, fibroblasts, and a variety of extracellular matrix components. This microenvironment plays a pivotal role in tumor initiation, progression, and metastasis. In bladder cancer, the tumor microenvironment is shaped by various immune cells, including tumor-infiltrating lymphocytes and suppressive populations like myeloid-derived suppressor cells and regulatory T cells, which can either enhance or dampen antitumor immune responses. These immune components play a pivotal role in disease progression and response to immunotherapies (9). Among the various components of the TME, extracellular vesicles (EVs) have emerged as critical mediators of intercellular communication in cancer biology (10). EVs are membrane-bound vesicles that can transport a variety of biomolecules, such as proteins, lipids, mRNA, and miRNA, between cells, thereby modulating the functions of recipient cells and influencing tumor biology. EVs are typically categorized into three primary subtypes based on their biogenesis mechanisms: exosomes, shed microvesicles, and apoptotic bodies. Each subtype reflects distinct mechanisms of formation and release, contributing to their diverse roles in cellular communication and immune modulation (11). In the context of bladder cancer, EVs have been implicated in various processes, including the promotion of tumor growth, angiogenesis, immune evasion, and the development of therapeutic resistance. The specific roles of EVs in bladder cancer suggest that they may serve as potential biomarkers for disease progression and therapeutic targets for novel treatment strategies. For instance, studies have shown that bladder cancer-derived EVs can carry tumor-specific markers, which could be harnessed for non-invasive diagnostics (12). Furthermore, the ability of EVs to deliver therapeutic agents selectively to tumor sites makes them an attractive platform for drug delivery systems (13).

This review aims to dissect the complex roles of EVs and assess their viability as diagnostic and therapeutic modalities in bladder cancer. Gaining insights into the mechanisms underlying EVs’ influence on bladder cancer biology is essential for devising cutting-edge strategies with the potential to transform patient care and propel forward the domain of bladder cancer therapeutics.

## 2 Extracellular vesicles in bladder cancer biology

**Figure 1:**
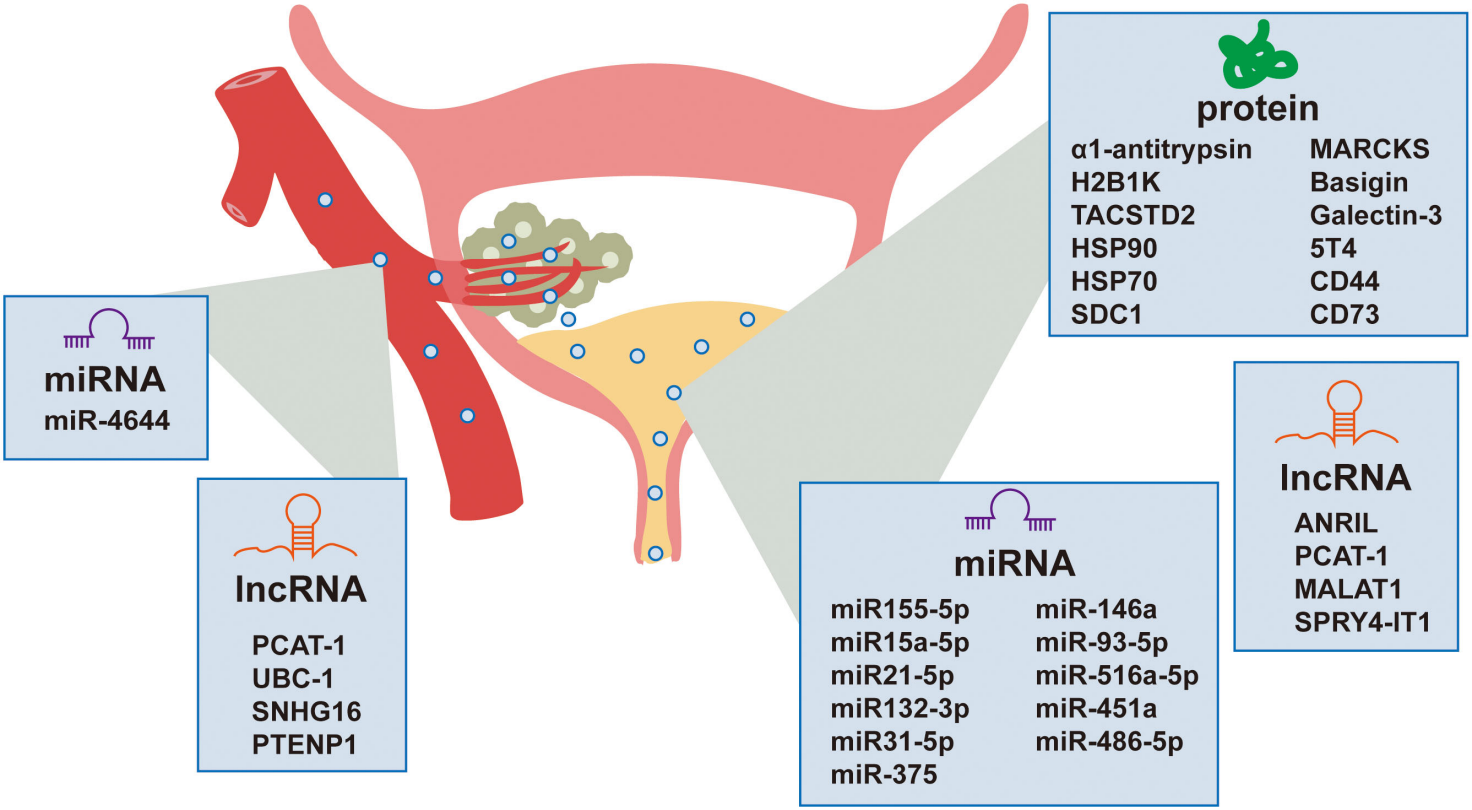
Extracellular vesicle-derived bladder cancer biomarkers in urine and blood.

The molecular heterogeneity of bladder cancer is captured within EVs, which are increasingly recognized for their role in reflecting the disease’s distinct molecular subtypes. These vesicles offer insights into the metabolic reprogramming and angiogenesis within the tumor microenvironment, as well as their capacity to modulate the behavior of recipient cells (**Figure 1**).

### 2.1 Molecular subtype reflection and communication

The molecular heterogeneity of bladder cancer is increasingly recognized as a critical factor influencing patient outcomes. EVs, particularly small extracellular vesicles (sEVs), have emerged as important mediators that reflect the molecular subtypes of bladder cancer, which are pivotal for prognosis and therapeutic response. Recent advancements have identified distinct mRNA-based molecular subtypes within bladder cancer, including luminal-papillary (LumP), luminal-nonspecified (LumNS), luminal-unstable (LumU), Stroma-rich, basal/squamous (Ba/Sq), and neuroendocrine-like (NE-like) (14). These subtypes exhibit unique EV cargo profiles that align with their biological behaviors. The stroma-rich subtype is characterized by marked stromal infiltration and activation of cancer-associated fibroblasts (CAFs). TGFβ is a central factor in CAF activation, promoting stromal remodeling and immunosuppression by inducing the differentiation of fibroblasts into a pro-tumorigenic phenotype. Ringuette Goulet C et al. elucidated the mechanism by which bladder cancer cells release exosomes containing TGFβ, which directly induce the differentiation of healthy fibroblasts into CAFs through SMAD pathway activation, highlighting exosomes as novel modulators of stromal cell differentiation and key contributors to the tumor microenvironment (15). Slabáková E et al. highlights the critical role of non-coding RNAs, particularly miRNAs, in the NE-like differentiation of prostate cancer, with exosomes serving as key mediators of these molecular changes (16). Similar to prostate cancer, the NE-like subtype of bladder cancer exhibits analogous molecular traits, with exosomes holding potential to capture these distinct molecular signatures.

The reflection of these molecular subtypes in sEVs is significant because it suggests that sEVs can serve as non-invasive biomarkers that mirror the molecular landscape of bladder cancer (17). This discovery is particularly relevant for liquid biopsy strategies, which aim to provide real-time molecular profiling of cancer through minimally invasive means. The concordance between the molecular subtype classification of formalin fixed paraffin embedded (FFPE) tumor tissues and their derived sEVs underscores the potential of sEVs in personalized medicine and precision oncology.

### 2.2 Metabolic reprogramming and angiogenesis

EVs facilitate metabolic reprogramming within the TME, particularly in the context of angiogenesis (18). In nutrient-deprived TMEs, bladder cancer cells are known to secrete glutamine fructose-6-phosphate transaminase 1 (GFAT1) via sEVs. This secretion reprograms glucose metabolism in endothelial cells (ECs), increasing the flux through the hexosamine biosynthesis pathway and enhancing O-linked N-acetylglucosamine (O-GlcNAc)ylation (19). This metabolic symbiosis between bladder cancer cells and ECs is crucial for promoting angiogenesis, which is a critical process in tumor growth and progression.

The modulation of metabolic pathways by EVs is not limited to glucose metabolism. EVs also participate in the reprogramming of lipid metabolism, which is essential for tumor growth and survival (20). For instance, EVs can transfer lipids and lipoproteins between cells, thereby influencing the lipid composition of recipient cells and potentially modulating their signaling pathways and metabolic activities (21). Additionally, EVs can carry metabolic enzymes and transporters that directly participate in metabolic reactions, further highlighting their role in metabolic reprogramming (22).

EVs play a critical role in tumor angiogenesis by transferring bioactive cargoes such as proteins, miRNAs, and lncRNAs to endothelial cells, promoting proliferation, migration, and tube formation (23). Tumor-derived EVs enhance angiogenesis through direct interactions with endothelial cells and via non-tumor cells like fibroblasts and macrophages (24, 25). EVs from cancer-associated fibroblasts (CAFs) and tumor-associated macrophages (TAMs) also contribute to angiogenesis by activating signaling pathways such as VEGF/VEGFR, JAK/STAT3, and Hippo. Li X et al. (19) highlighted that bladder cancer-derived sEVs carrying glutamine-fructose-6-phosphate aminotransferase 1 (GFAT1) enhance tumor angiogenesis by reprogramming glucose metabolism in endothelial cells through the hexosamine biosynthesis pathway (HBP), leading to increased O-GlcNAcylation.

Understanding the metabolic reprogramming mediated by EVs provides a new perspective on the complex interplay between cancer cells and their microenvironment. Targeting the metabolic pathways modulated by EVs could offer a novel therapeutic strategy for disrupting the pro-angiogenic activities within the TME, thereby inhibiting tumor growth and metastasis (26).

### 2.3 Modulation of cellular behavior

sEVs are increasingly recognized for their ability to modulate the behavior of recipient cells within the TME. This modulation is crucial in the context of bladder cancer, where sEVs from non-stem cancer cells (NSCCs) have been shown to significantly alter the properties of cancer stem cells (CSCs) (27). Specifically, these EVs can enhance the chemoresistance, aggressiveness, and self-renewal capabilities of CSCs, which are key determinants of tumor recurrence and therapeutic failure (28).

The interaction between NSCCs and CSCs through EVs is a critical communication mechanism that promotes disease progression and the acquisition of chemoresistance in bladder cancer (26). This finding challenges the traditional view of EVs as mere bystanders in cancer biology, positioning them instead as active participants in the complex cellular interactions that drive cancer progression (29). The ability of EVs to transfer functional proteins and RNAs between cells suggests a mechanism by which the TME can be reprogrammed to support tumor survival and resistance to therapy. Recent research has highlighted the role of exosomal long noncoding RNAs (lncRNAs) in modulating the TME, particularly in the context of lymphatic metastasis. Chen et al. (30) revealed that exosome-derived lncRNA ELNAT1 promotes lymphangiogenesis and lymph node metastasis in bladder cancer through SUMOylation-dependent pathways, underscoring the potential of targeting exosomal lncRNAs for therapeutic intervention in aggressive bladder cancer subtypes. Song et al. (31) provided a comprehensive analysis of the role of bladder cancer-derived exosomal KRT6B in cancer progression. It reveals that KRT6B, associated with epithelial-mesenchymal transition (EMT) and immune response, could serve as a significant prognostic marker and therapeutic target in bladder cancer.

Moreover, the modulation of CSC properties by NSCC-derived EVs is not limited to chemoresistance. EVs have also been implicated in the regulation of CSC plasticity, which is the ability of these cells to switch between stem-like and non-stem-like states, thereby contributing to tumor heterogeneity and adaptive responses to therapy (32). Chen Yang et al. (33) discovered that exosome-derived circTRPS1 in bladder cancer promotes tumor aggressiveness and CD8+ T cell exhaustion. This circRNA sponges miR-141-3p, modulating GLS1-mediated glutamine metabolism and intracellular ROS balance. The precise molecular mechanisms underlying these effects are still being elucidated, but it is clear that EVs play a multifaceted role in the TME, influencing both cellular behavior and the broader tumor microenvironment (34).

## 3 Diagnostic and prognostic potential of urinary and serum extracellular vesicles

### 3.1 Diagnostic potential of urinary and serum extracellular vesicles in bladder cancer

**Figure 2:**
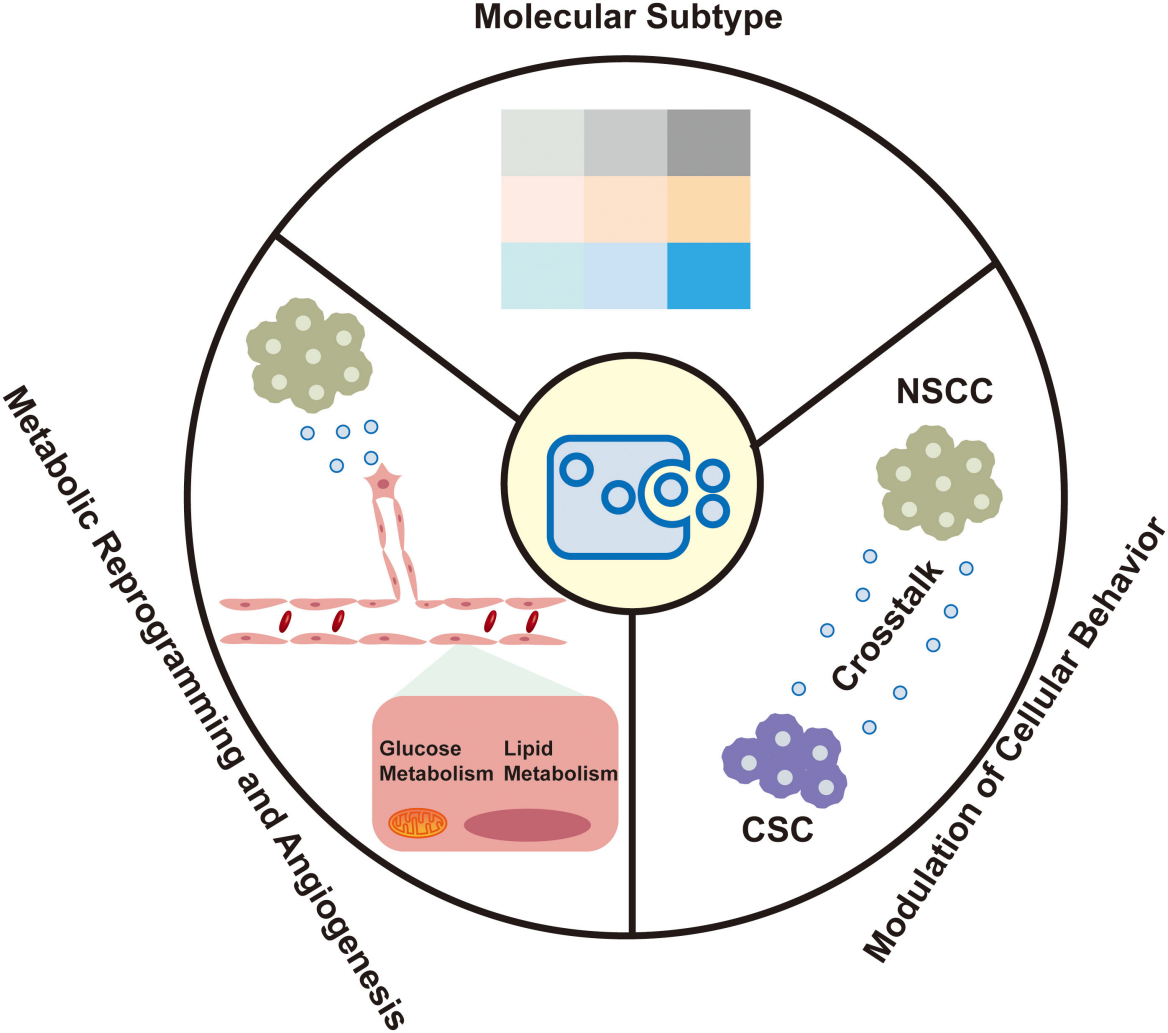
Extracellular vesicles in bladder cancer biology.

Non-invasive detection of bladder cancer using urinary and serum EVs has gained significant attention due to their accessibility and their ability to reflect the molecular landscape of the TME. Urinary EVs, which are nano-sized vesicles secreted by various cells including bladder cancer cells, can be isolated from urine samples and carry a cargo of proteins, nucleic acids, and lipids that can serve as biomarkers for bladder cancer. Recent studies have identified multiple EV-based biomarkers with clinical relevance and their diagnostic performance parameters are systematically summarized in **Table 1**. The non-invasive nature of urine collection makes urinary EVs an attractive source for diagnostic biomarkers. Additionally, serum EVs also hold significant potential as biomarkers for bladder cancer, offering a promising avenue for diagnosis (**Figure 2**).

**Table 1:** Summary of diagnostic biomarkers in bladder cancer using EVs.

Biomarker Type	Biomarker Name	Source	Age (Range)	Gender (M/F Ratio)	Technique Used	Sensitivity (%)	Specificity (%)	Reference
Protein	alpha 1-antitrypsin histone H2B1K	Urine	67.34 ± 11.35	1.93	MALDI-TOF spectrometry	62.70	87.59	(35)
	TACSTD2	Urine	66.88 ± 12.65	2.33	LC−MRM/MS Elisa	73.60	76.50	(36)
	HSP90 SDC1 MARCKS	Urine	71 (31-­87)	2.64	SRM/MRM analysis	82.50 82.50 60.50	70.00 63.30 80.00	(37)
	HSP70 HSP90 Basigin Galectin-3 5T4 CD44 CD73	Urine	N/A	N/A	Western blotting, flotation on linear sucrose gradients, flow cytometry	N/A	N/A	(38)
miRNA	miR-4644	Serum	62.10 ± 4.02	3.75	miRNA microarray analysis	N/A	N/A	(39)
	miR21-5p miR155-5p miR15a-5p miR132-3p miR31-5p	Urine	70.5 (47–91)	4.14	NanoSight™ particle tracking analysis, Microarray analysis for miRNAs	75.00	95.80	(40)
	miR-375 miR-146a	Urine	79.5 ± 10.30 66.94 ± 11.62	17.00 4.33	RNA isolation and microarray detection, Reverse transcription and real-time qPCR	N/A	N/A	(41)
	miR-93-5p miR-516a-5p	Urine	62.25 ± 2.91	1.00	quantitative reverse-transcription polymerase chain reaction assay	74.10 72.90	90.20 89.90	(42)
	miR-451a miR-486-5p	Urine	72.37 ± 8.68	0.28	exoRNeasy kit, miRNeasy kit	N/A	N/A	(43)
lncRNA	PCAT-1 UBC-1 SNHG16	Serum	N/A	N/A	Nanoparticle tracking, Western blotting, RNA extraction and reverse	N/A	N/A	(44)
	ANRIL PCAT-1	Urine	55.84 ± 11.96	N/A	Exosomal RNA isolation, Quantitative real-time PCR analysis	46.67 43.33	87.50 87.50	(45)
	MALAT1 PCAT-1 SPRY4-IT1	Urine	N/A	N/A	TEM, Western blotting analysis, nanoparticle tracking analysis (NTA), flow cytometry	72.10 72.10 66.30	84.60 81.70 76.90	(46)
	PTENP1	Serum	67.0 ± 9.8	2.57	TEM, Western blots, RNA isolation, quantitative real-time PCR	65.40	84.20	(47)

### 3.2 Proteomic analysis and identification of specific biomarkers

Proteomic analysis of urinary EVs has identified specific proteins that are differentially expressed in bladder cancer patients compared to healthy controls. Lin et al. (35) utilized MALDI-TOF spectrometry to analyze urinary exosomes and identified alpha 1-antitrypsin and H2B1K as potential diagnostic and prognostic biomarkers for urothelial carcinoma. C. L. Chen et al. (36) identified 107 differentially expressed proteins as potential noninvasive biomarkers, among which TACSTD2 showed significant association with bladder cancer and was validated as a promising diagnostic marker. A study by E. Tomiyama et al. (37) employed proteomic analysis of urinary and tissue-exudative EVs to uncover potential biomarkers for bladder cancer, revealing that a combination of these analyses could selectively detect cancer-specific EVs. The research identified six urinary EV proteins that were significantly elevated in bladder cancer patients, with HSP90, SDC1, and MARCKS being the most upregulated. Additionally, the study by Welton et al. identified 353 proteins in bladder cancer-derived exosomes, including members of the ESCRT family, heat shock proteins (hsp70, hsp90), cytoskeletal elements, and a wide array of transmembrane proteins (38).

### 3.3 MicroRNAs as biomarkers

MicroRNAs (miRNAs), small non-coding RNAs that play crucial roles in gene regulation, have emerged as potential biomarkers for bladder cancer diagnosis. Certain miRNAs have been identified as potential biomarkers due to their increased presence in urinary EVs from patients with bladder cancer. Matsuzaki et al. identified five miRNAs (miR155-5p, miR15a-5p, miR21-5p, miR132-3p, and miR31-5p) in urinary EVs, with miR-21-5p showing considerable sensitivity (75%) and specificity (98%) for bladder cancer diagnosis (40). These miRNAs could be particularly useful in early disease diagnosis, even in cases with false-negative cytology results. Andreu et al. (41) conducted a study in which they isolated urinary EVs from both healthy individuals and patients with low or high-grade bladder cancer. They analyzed the protein and microRNA (miRNA) profiles of these EVs and discovered that miR-375 and miR-146a serve as significant diagnostic biomarkers for high-grade and low-grade bladder cancer, respectively. This finding underscores the promising potential of these markers in the field of precision diagnostics. H. Lin et al. (42) identified urinary exosomal miRNAs, particularly miR-93-5p and miR-516a-5p, as potential non-invasive biomarkers for diagnosing bladder cancer, with miR-93-5p showing a significant increase in muscle-invasive bladder cancer. The research also revealed that miR-93-5p promotes bladder cancer cell proliferation, invasion, and migration by targeting and suppressing the tumor suppressor gene BTG2. O. Strømme et al. (43) investigated differentially expressed extracellular vesicle-contained microRNAs in urine and serum before and after transurethral resection of bladder tumors, identifying miR-451a and miR-486-5p as potential biomarkers for recurrence-free survival in patients with stage T1 bladder cancer. L. Yan et al. (39) identified miR-4644 as an upregulated microRNA in plasma exosomes of bladder cancer patients, which promoted cancer progression by targeting UBIAD1.

### 3.4 Long non-coding RNAs as emerging biomarkers

Long non-coding RNAs (lncRNAs) have also been implicated in bladder cancer biology. Specific lncRNAs, such as PCAT-1, UBC-1, and SNHG16, have been found to be significantly increased in the serum of bladder cancer patients, suggesting their potential as diagnostic markers (44). M. Abbastabar et al. (45) investigated the potential of urinary exosomal lncRNAs PVT-1, ANRIL, and PCAT-1 as diagnostic biomarkers for bladder cancer, finding significantly higher expression of ANRIL and PCAT-1 in bladder cancer patients compared to healthy controls. The diagnostic accuracy of these lncRNAs, as measured by the area under the ROC curve (AUC), indicates their potential utility in distinguishing bladder cancer from healthy individuals. Y. Zhan et al. (46) developed a urinary exosome-derived lncRNA panel consisting of MALAT1, PCAT-1, and SPRY4-IT1 for diagnosing bladder cancer, demonstrating high diagnostic accuracy compared to urine cytology. Additionally, the upregulation of PCAT-1 and MALAT1 was associated with poor recurrence-free survival in non-muscle-invasive bladder cancer, indicating the potential of these lncRNAs as prognostic factors. R. Zheng et al. (47) identified exosomal long non-coding RNA PTENP1 as a potential biomarker for bladder cancer, demonstrating that it is downregulated in cancer tissues and plasma exosomes, and that it can suppress bladder cancer progression by competitively binding to microRNA-17 and modulating PTEN expression. The study suggests that exosomal PTENP1 from normal cells can be transferred to bladder cancer cells, reducing their malignant behavior both *in vitro* and *in vivo*.

### 3.5 EV biomarkers for prognosis, and prediction of therapeutic response

EV biomarkers have shown potential in predicting patient outcomes and survival rates in bladder cancer. Specific EV-associated proteins and miRNAs can indicate disease severity and progression. For instance, a study by Lin et al. identified alpha-1 antitrypsin and H2B1K as prognostic biomarkers in bladder cancer, associated with higher grade tumors and potentially poorer outcomes (35). Additionally, miRNAs such as miR-21-5p have been found to correlate with disease stage and aggressiveness, providing insights into patient prognosis (40). Moreover, certain EV biomarkers have been associated with tumor aggressiveness and patient survival outcomes, underscoring their importance in prognostic evaluations (48).

The predictive power of EV biomarkers extends to assessing responses to chemotherapy and immunotherapy. Changes in EV cargo before and after treatment can indicate whether a treatment is effective. A study by Hiltbrunner et al. demonstrated that urinary exosomes from bladder cancer patients retained a cancer phenotype even after complete pathological downstaging, suggesting their role in predicting therapeutic response and potential recurrence (49). This finding underscores the importance of EVs in personalizing treatment strategies and monitoring treatment efficacy.

### 3.6 Comparative analysis of EV biomarkers with traditional diagnostic methods

Comparative analyses between EV biomarkers and traditional diagnostic methods, such as cystoscopy and urinary cytology, have demonstrated the potential superiority of EV-based biomarkers in terms of sensitivity and specificity for bladder cancer detection. EVs can provide molecular insights that are not accessible through conventional methods, offering a more comprehensive understanding of the disease state. Moreover, the non-invasive nature of EV biomarker detection makes them particularly attractive for routine monitoring and repeated assessments, which are often required in the management of bladder cancer.

However, despite the advantages of EV biomarkers, there are challenges to their implementation in clinical diagnostics. The isolation and analysis of EVs require standardized procedures to ensure reproducibility and comparability of results. Current methods, such as ultracentrifugation and immunoaffinity-based separation, can be labor-intensive and may not yield pure EV populations due to the presence of soluble proteins and other contaminants. Furthermore, the functional roles of many EV biomarkers in bladder cancer are yet to be fully elucidated. While traditional diagnostic methods like cystoscopy provide direct visualization of tumors, EV biomarkers offer a molecular insight that complements these techniques, potentially increasing the sensitivity of cytology, especially for low-grade tumors that are less likely to exfoliate cells. The integration of EV biomarkers with existing diagnostic methods could lead to a more personalized and precise approach to bladder cancer management.

## 4 Therapeutic strategies utilizing extracellular vesicles

EVs have emerged as natural nanocarriers for drug delivery in bladder cancer treatment due to their unique biological properties. The lipid bilayer of EVs allows for the encapsulation and protection of therapeutic agents, including small molecule drugs, siRNAs, miRNAs, and proteins. This feature is particularly advantageous as it enables the delivery of a wide range of therapeutics that are otherwise challenging to administer due to their susceptibility to degradation or rapid clearance from the body (50–52). Recent studies have demonstrated the potential of EVs to carry chemotherapeutic agents, such as doxorubicin, resulting in significant tumor volume reduction and prolonged survival in preclinical models (52).

Among the emerging therapeutic markers, miRNAs and targeted signaling pathways stand out as the most robust candidates based on mechanistic depth and preclinical validation. In bladder cancer therapeutics, the innovative utilization of EVs has been exemplified by studies targeting key oncogenic pathways. Cai et al. (53) illuminated the role of exosome-transmitted microRNA-133b (miR-133b) in inhibiting bladder cancer proliferation. They discovered that miR-133b, which is downregulated in bladder cancer, could be packaged into exosomes to mediate communication between tumor cells, thereby affecting their proliferation and apoptosis. The study revealed that exosomal miR-133b significantly reduced the viability and increased apoptosis in bladder cancer cells by upregulating dual-specificity protein phosphatase 1 (DUSP1), a key regulator of cell proliferation and apoptosis. This finding suggests that exosomal miR-133b could serve as a novel therapeutic agent by modulating intracellular communication and suppressing bladder cancer growth. Li et al. (54) explored the suppressive role of exosomal miR-375-3p in bladder cancer via the Wnt/β-catenin pathway. They reported that miR-375-3p, another downregulated miRNA in BC, directly targets Frizzled-8 (FZD8), a key receptor in the Wnt signaling pathway. By inhibiting FZD8, miR-375-3p blocks the Wnt/β-catenin pathway, leading to the suppression of BC cell proliferation and metastasis, and the promotion of apoptosis. The study demonstrated that miR-375-3p overexpression in a T24 xenograft mouse model resulted in reduced tumor volume and Ki67 proliferation index, indicating its potential as a therapeutic strategy for bladder cancer.

Artificial circular RNAs (acircRNAs) targeting β-catenin and NF-κB represent a novel but less validated approach. Zhou et al. (55) recently reported on the development of acircRNAs delivered via exosomes for bladder cancer gene therapy. These acircRNAs, designed to target β-catenin and NF-κB, were effectively encapsulated into exosomes using a CD63-HuR fusion protein, leading to suppressed proliferation and enhanced apoptosis in bladder cancer cells. This approach demonstrates the promise of exosome-mediated gene therapy in treating bladder cancer by specifically targeting key oncogenic pathways.

PLK-1 siRNA delivered via exosomes exemplifies the potential of RNAi-based therapies. K. A. Greco et al. (56) found that bladder cancer cells internalize more exosomes than normal urothelial cells, and that exosomes can effectively deliver PLK-1 siRNA, leading to gene silencing and reduced cell proliferation. PLK-1’s essential role in mitosis and its overexpression in aggressive subtypes make it a compelling target for combination therapies.

For NMIBC, tumor-derived microparticles (T-MPs) may offer immediate clinical utility. X. Jin et al. (57) demonstrated that pre-instillation of T-MPs could enhance the efficacy of intravesical chemotherapy in NMIBC by increasing drug retention and promoting drug entry into cancer cell nuclei through a lysosomal pathway. The findings suggest that T-MPs could serve as a potent sensitizer to augment NMIBC chemotherapy with significant clinical benefits.

These studies underscore the therapeutic potential of EVs-based strategies in bladder cancer.

## 5 Challenges and future directions

EVs are heterogeneous in nature, which poses a significant challenge in their isolation (58). Traditional methods for EV isolation, such as ultracentrifugation, may not be sufficient to separate all subpopulations effectively. Moreover, the overlap in size and density between exosomes and microvesicles complicates their distinction. Advanced techniques like acoustic purification and acoustofluidic separation are emerging but require further validation and standardization (59). A recent study presented the synthesis of magnetic 3D ordered macroporous zeolitic imidazolate framework-8 (magMZIF-8) for efficient isolation of urinary exosomes, which, combined with LC-MS/MS and machine learning algorithms, showed potential as a reliable diagnostic tool for early-stage bladder cancer. The magMZIF-8 material enables rapid and high-purity exosome isolation, facilitating the detection of differential metabolites that can accurately differentiate and predict bladder cancer (60).

The characterization of EVs is another technical challenge due to their diverse molecular content, which includes proteins, nucleic acids, and lipids. The need for sensitive and specific detection methods is crucial, as the cargo of EVs can be stabilized and protected from extracellular conditions, making them attractive for diagnostic purposes. However, the heterogeneity of EVs makes it difficult to standardize characterization methods (61).

The transition from bench to bedside is hampered by the lack of standardized protocols and the need for more robust clinical validation. The potential of EVs as minimally invasive liquid biopsies has accelerated research, but there is a need to validate potential biomarkers in physiologically relevant biofluids. Additionally, the economic impact of bladder cancer necessitates effective intervention strategies and resource allocation, which are currently limited by the challenges in EV analysis.

The heterogeneity of EVs and the variety of methods used for their isolation and characterization highlight the urgent need for standardized protocols in bladder cancer research. The International Society for Extracellular Vesicles (ISEV) has made recommendations for the nomenclature and isolation of EVs, which is a step towards standardization (62). However, more work is needed to establish universally accepted protocols that can be applied across different research settings and clinical trials. Future research should focus on the development of multi-biomarker panels for bladder cancer. Encapsulated miRNA in EVs may boost tumor invasiveness and the dissemination of metastatic cells through intercellular communication, making them promising candidates for multi-biomarker panels (63). Clinical trials are essential for the validation of EV-based biomarkers and therapeutic strategies. There is a need for more robust clinical studies to establish the efficacy of EV-based diagnostics and therapeutics in bladder cancer management.

## 6 Conclusions

In conclusion, EVs offer a multifaceted approach to bladder cancer management, serving as both biomarkers for diagnosis, prognosis, and therapeutic response prediction, and as therapeutic agents for drug delivery. The ability of EVs to carry specific cargo, including proteins, nucleic acids, and lipids, positions them as promising tools in liquid biopsies and personalized oncology. However, the heterogeneity of EVs and the lack of standardized isolation and characterization methods present challenges that must be addressed through further research and clinical trials. The development of multi-biomarker panels and the integration of EV-based diagnostics with traditional methods could lead to more precise and personalized bladder cancer management strategies. As the field advances, the focus should be on establishing universally accepted protocols and conducting large-scale clinical studies to validate the efficacy of EV-based diagnostics and therapeutics, ultimately aiming to improve patient outcomes and reduce the global burden of bladder cancer.

## References

[1] Sung H Ferlay J Siegel RL Laversanne M Soerjomataram I Jemal A. Global cancer statistics 2020: globocan estimates of incidence and mortality worldwide for 36 cancers in 185 countries. CA Cancer J Clin. (2021) 71:209–49. doi: 10.3322/caac.21660 33538338

[2] Siegel RL Giaquinto AN Jemal A. Cancer statistics, 2024. CA Cancer J Clin. (2024) 74:12–49. doi: 10.3322/caac.21820 38230766

[3] Antoni S Ferlay J Soerjomataram I Znaor A Jemal A Bray F. Bladder cancer incidence and mortality: A global overview and recent trends. Eur Urol. (2017) 71:96–108. doi: 10.1016/j.eururo.2016.06.010 27370177

[4] Kates M Date A Yoshida T Afzal U Kanvinde P Babu T. Preclinical evaluation of intravesical cisplatin nanoparticles for non-muscle-invasive bladder cancer. Clin Cancer Res. (2017) 23:6592–601. doi: 10.1158/1078-0432.Ccr-17-1082 PMC6487844 28808039

[5] Babjuk M Burger M Comperku EM Gontero P Mostafid AH Palou J. European association of urology guidelines on non-muscle-invasive bladder cancer (Tat1 and carcinoma in situ) - 2019 update. Eur Urol. (2019) 76:639–57. doi: 10.1016/j.eururo.2019.08.016 31443960

[6] Witjes JA Bruins HM Cathomas R Compomas EM Cowan NC Gakis G. European association of urology guidelines on muscle-invasive and metastatic bladder cancer: summary of the 2020 guidelines. Eur Urol. (2021) 79:82–104. doi: 10.1016/j.eururo.2020.03.055 32360052

[7] Babjuk M Burger M Capoun O Cohen D Compnnku EM Dominguez Escrig JL. European association of urology guidelines on non-muscle-invasive bladder cancer (Ta, T1, and carcinoma in situ). Eur Urol. (2022) 81:75–94. doi: 10.1016/j.eururo.2021.08.010 34511303

[8] Robertson AG Kim J Al-Ahmadie H Bellmunt J Guo G Cherniack AD. Comprehensive molecular characterization of muscle-invasive bladder cancer. Cell. (2017) 171:540–56.e25. doi: 10.1016/j.cell.2017.09.007 28988769 PMC5687509

[9] Tran L Xiao JF Agarwal N Duex JE Theodorescu D. Advances in bladder cancer biology and therapy. Nat Rev Cancer. (2021) 21:104–21. doi: 10.1038/s41568-020-00313-1 PMC10112195 33268841

[10] Shehzad A Islam SU Shahzad R Khan S Lee YS. Extracellular vesicles in cancer diagnostics and therapeutics. Pharmacol Ther. (2021) 223:107806. doi: 10.1016/j.pharmthera.2021.107806 33465400

[11] Marar C Starich B Wirtz D. Extracellular vesicles in immunomodulation and tumor progression. Nat Immunol. (2021) 22:560–70. doi: 10.1038/s41590-021-00899-0 PMC9389600 33753940

[12] Li SR Man QW Gao X Lin H Wang J Su FC. Tissue-derived extracellular vesicles in cancers and non-cancer diseases: present and future. J Extracell Vesicles. (2021) 10:e12175. doi: 10.1002/jev2.12175 34918479 PMC8678102

[13] Zhang X Zhang H Gu J Zhang J Shi H Qian H. Engineered extracellular vesicles for cancer therapy. Adv Mater. (2021) 33:e2005709. doi: 10.1002/adma.202005709 33644908

[14] The Cancer Genome Atlas Research Network. Comprehensive molecular characterization of urothelial bladder carcinoma. Nature. (2014) 507:315–22. doi: 10.1038/nature12965 PMC3962515 24476821

[15] Ringuette Goulet C Bernard G Tremblay S Chabaud S Bolduc S Pouliot F. Exosomes Induce Fibroblast Differentiation into Cancer-Associated Fibroblasts through Tgfo Signaling. Mol Cancer Res. (2018) 16:1196–204. doi: 10.1158/1541-7786.Mcr-17-0784 29636362

[16] Slabblab E Kahounov Z Prochnov J Souček K. Regulation of neuroendocrine-like differentiation in prostate cancer by non-coding rnas. Noncod RNA. (2021) 7(4):75. doi: 10.3390/ncrna7040075 PMC8704250 34940756

[17] Dong L Feng M Kuczler MD Horie K Kim CJ Ma Z. Tumour tissue-derived small extracellular vesicles reflect molecular subtypes of bladder cancer. J Extracell Vesicles. (2024) 13:e12402. doi: 10.1002/jev2.12402 38293707 PMC10828726

[18] Grange C Tapparo M Collino F Vitillo L Damasco C Deregibus MC. Microvesicles released from human renal cancer stem cells stimulate angiogenesis and formation of lung premetastatic niche. Cancer Res. (2011) 71:5346–56. doi: 10.1158/0008-5472.Can-11-0241 21670082

[19] Li X Peng X Zhang C Bai X Li Y Chen G. Bladder cancer-derived small extracellular vesicles promote tumor angiogenesis by inducing hbp-related metabolic reprogramming and serrs O-glcnacylation in endothelial cells. Adv Sci (Weinh). (2022) 9:e2202993. doi: 10.1002/advs.202202993 36045101 PMC9596856

[20] PollPol B Xavier CPR Kopecka J Riganti C Vasconcelos MH. The role of extracellular vesicles in glycolytic and lipid metabolic reprogramming of cancer cells: consequences for drug resistance. Cytok Growth Factor Rev. (2023) 73:150–62. doi: 10.1016/j.cytogfr.2023.05.001 37225643

[21] Menard JA Cerezo-Magaña M Belting M. Functional role of extracellular vesicles and lipoproteins in the tumour microenvironment. Philos Trans R Soc Lond B Biol Sci. (2018) 373(1737):20160480. doi: 10.1098/rstb.2016.0480 29158310 PMC5717435

[22] Minic Z Li Y Hüttmann N Uppal GK D’Mello R Berezovski MV. Lysine acetylome of breast cancer-derived small extracellular vesicles reveals specific acetylation patterns for metabolic enzymes. Biomedicines. (2023) 11(4):1076. doi: 10.3390/biomedicines11041076 37189694 PMC10135746

[23] Ye ZW Yu ZL Chen G Jia J. Extracellular vesicles in tumor angiogenesis and resistance to anti-angiogenic therapy. Cancer Sci. (2023) 114:2739–49. doi: 10.1111/cas.15801 PMC10323098 37010195

[24] Zhou X Yan T Huang C Xu Z Wang L Jiang E. Melanoma cell-secreted exosomal mir-155-5p induce proangiogenic switch of cancer-associated fibroblasts via socs1/jak2/stat3 signaling pathway. J Exp Clin Cancer Res. (2018) 37:242. doi: 10.1186/s13046-018-0911-3 30285793 PMC6169013

[25] Ludwig N Rubenich DS Zaręba Ł Siewiera J Pieper J Braganhol E. Potential roles of tumor cell- and stroma cell-derived small extracellular vesicles in promoting a pro-angiogenic tumor microenvironment. Cancers (Basel). (2020) 12(10):6277–6289. doi: 10.3390/cancers12123599 PMC7760552 33276428

[26] Melo SA Sugimoto H O’Connell JT Kato N Villanueva A Vidal A. Cancer exosomes perform cell-independent microrna biogenesis and promote tumorigenesis. Cancer Cell. (2014) 26:707–21. doi: 10.1016/j.ccell.2014.09.005 PMC4254633 25446899

[27] Chung WM Molony RD Lee YF. Non-stem bladder cancer cell-derived extracellular vesicles promote cancer stem cell survival in response to chemotherapy. Stem Cell Res Ther. (2021) 12:533. doi: 10.1186/s13287-021-02600-6 34627375 PMC8502272

[28] Lin Z Wu Y Xu Y Li G Li Z Liu T. Mesenchymal stem cell-derived exosomes in cancer therapy resistance: recent advances and therapeutic potential. Mol Cancer. (2022) 21:179. doi: 10.1186/s12943-022-01650-5 36100944 PMC9468526

[29] Paskeh MDA Entezari M Mirzaei S Zabolian A Saleki H Naghdi MJ. Emerging role of exosomes in cancer progression and tumor microenvironment remodeling. J Hematol Oncol. (2022) 15:83. doi: 10.1186/s13045-022-01305-4 35765040 PMC9238168

[30] Chen C Zheng H Luo Y Kong Y An M Li Y. Sumoylation promotes extracellular vesicle-mediated transmission of lncrna elnat1 and lymph node metastasis in bladder cancer. J Clin Invest. (2021) 131(8):e146431. doi: 10.1172/jci146431 33661764 PMC8262506

[31] Song Q Yu H Cheng Y Han J Li K Zhuang J. Bladder cancer-derived exosomal krt6b promotes invasion and metastasis by inducing emt and regulating the immune microenvironment. J Transl Med. (2022) 20:308. doi: 10.1186/s12967-022-03508-2 35794606 PMC9258227

[32] Mani SA Guo W Liao MJ Eaton EN Ayyanan A Zhou AY. The epithelial-mesenchymal transition generates cells with properties of stem cells. Cell. (2008) 133:704–15. doi: 10.1016/j.cell.2008.03.027 PMC2728032 18485877

[33] Yang C Wu S Mou Z Zhou Q Dai X Ou Y. Exosome-derived circtrps1 promotes Malignant phenotype and cd8+ T cell exhaustion in bladder cancer microenvironments. Mol Ther. (2022) 30:1054–70. doi: 10.1016/j.ymthe.2022.01.022 PMC8899700 35038580

[34] Peinado H Alečković M Lavotshkin S Matei I Costa-Silva B Moreno-Bueno G. Melanoma Exosomes Educate Bone Marrow Progenitor Cells toward a Pro-Metastatic Phenotype through Met. Nat Med. (2012) 18:883–91. doi: 10.1038/nm.2753 PMC3645291 22635005

[35] Lin SY Chang CH Wu HC Lin CC Chang KP Yang CR. Proteome profiling of urinary exosomes identifies alpha 1-antitrypsin and H2b1k as diagnostic and prognostic biomarkers for urothelial carcinoma. Sci Rep. (2016) 6:34446. doi: 10.1038/srep34446 27686150 PMC5043375

[36] Chen CL Lai YF Tang P Chien KY Yu JS Tsai CH. Comparative and targeted proteomic analyses of urinary microparticles from bladder cancer and hernia patients. J Proteome Res. (2012) 11:5611–29. doi: 10.1021/pr3008732 23082778

[37] Tomiyama E Matsuzaki K Fujita K Shiromizu T Narumi R Jingushi K. Proteomic analysis of urinary and tissue-exudative extracellular vesicles to discover novel bladder cancer biomarkers. Cancer Sci. (2021) 112:2033–45. doi: 10.1111/cas.14881 PMC8088963 33721374

[38] Welton JL Khanna S Giles PJ Brennan P Brewis IA Staffurth J. Proteomics analysis of bladder cancer exosomes. Mol Cell Proteomics. (2010) 9:1324–38. doi: 10.1074/mcp.M000063-MCP201 PMC2877990 20224111

[39] Yan L Li Q Sun K Jiang F. Mir-4644 is upregulated in plasma exosomes of bladder cancer patients and promotes bladder cancer progression by targeting ubiad1. Am J Transl Res. (2020) 12:6277–89. PMC7653622 33194029

[40] Matsuzaki K Fujita K Jingushi K Kawashima A Ujike T Nagahara A. Mir-21-5p in urinary extracellular vesicles is a novel biomarker of urothelial carcinoma. Oncotarget. (2017) 8:24668–78. doi: 10.18632/oncotarget.14969 PMC5421878 28160564

[41] Andreu Z Otta Oshiro R Redruello A López-Martín S Gutitellouncotarg C Morato E. Extracellular vesicles as a source for non-invasive biomarkers in bladder cancer progression. Eur J Pharm Sci. (2017) 98:70–9. doi: 10.1016/j.ejps.2016.10.008 27751843

[42] Lin H Shi X Li H Hui J Liu R Chen Z. Urinary exosomal mirnas as biomarkers of bladder cancer and experimental verification of mechanism of mir-93-5p in bladder cancer. BMC Cancer. (2021) 21:1293. doi: 10.1186/s12885-021-08926-x 34861847 PMC8641206

[43] Strømme O Heck KA Brede G Lindholm HT Otterlei M Arum CJ. Differentially Expressed Extracellular Vesicle-Contained Micrornas before and after Transurethral Resection of Bladder Tumors. Curr Issues Mol Biol. (2021) 43:286–300. doi: 10.3390/cimb43010024 34199766 PMC8929081

[44] Zhang S Du L Wang L Jiang X Zhan Y Li J. Evaluation of serum exosomal lncrna-based biomarker panel for diagnosis and recurrence prediction of bladder cancer. J Cell Mol Med. (2019) 23:1396–405. doi: 10.1111/jcmm.14042 PMC6349164 30467945

[45] Abbastabar M Sarfi M Golestani A Karimi A Pourmand G Khalili E. Tumor-derived urinary exosomal long non-coding rnas as diagnostic biomarkers for bladder cancer. Excli J. (2020) 19:301–10. doi: 10.17179/excli2019-1683 PMC7104196 32231490

[46] Zhan Y Du L Wang L Jiang X Zhang S Li J. Expression signatures of exosomal long non-coding rnas in urine serve as novel non-invasive biomarkers for diagnosis and recurrence prediction of bladder cancer. Mol Cancer. (2018) 17:142. doi: 10.1186/s12943-018-0893-y 30268126 PMC6162963

[47] Zheng R Du M Wang X Xu W Liang J Wang W. Exosome-transmitted long non-coding rna ptenp1 suppresses bladder cancer progression. Mol Cancer. (2018) 17:143. doi: 10.1186/s12943-018-0880-3 30285771 PMC6169076

[48] Tong Y Liu X Xia D Peng E Yang X Liu H. Biological roles and clinical significance of exosome-derived noncoding rnas in bladder cancer. Front Oncol. (2021) 11:704703. doi: 10.3389/fonc.2021.704703 34692482 PMC8530185

[49] Hiltbrunner S Mints M Eldh M Rosenblatt R Holmström B Alamdari F. Urinary exosomes from bladder cancer patients show a residual cancer phenotype despite complete pathological downstaging. Sci Rep. (2020) 10:5960. doi: 10.1038/s41598-020-62753-x 32249794 PMC7136268

[50] Dai J Su Y Zhong S Cong L Liu B Yang J. Exosomes: key players in cancer and potential therapeutic strategy. Signal Transduct Target Ther. (2020) 5:145. doi: 10.1038/s41392-020-00261-0 32759948 PMC7406508

[51] Barile L Vassalli G. Exosomes: therapy delivery tools and biomarkers of diseases. Pharmacol Ther. (2017) 174:63–78. doi: 10.1016/j.pharmthera.2017.02.020 28202367

[52] Yong T Zhang X Bie N Zhang H Zhang X Li F. Tumor exosome-based nanoparticles are efficient drug carriers for chemotherapy. Nat Commun. (2019) 10:3838. doi: 10.1038/s41467-019-11718-4 31444335 PMC6707218

[53] Cai X Qu L Yang J Xu J Sun L Wei X. Exosome-transmitted microrna-133b inhibited bladder cancer proliferation by upregulating dual-specificity protein phosphatase 1. Cancer Med. (2020) 9:6009–19. doi: 10.1002/cam4.3263 PMC7433806 32627968

[54] Li Q Huyan T Cai S Huang Q Zhang M Peng H. The role of exosomal mir-375-3p: A potential suppressor in bladder cancer via the wnt/nt/errsor pathway. FASEB J. (2020) 34:12177–96. doi: 10.1096/fj.202000347R 32716585

[55] Zhou Q Fang L Tang Y Wang Q Tang X Zhu L. Exosome-mediated delivery of artificial circular rnas for gene therapy of bladder cancer. J Cancer. (2024) 15:1770–8. doi: 10.7150/jca.90620 PMC10869980 38370378

[56] Greco KA Franzen CA Foreman KE Flanigan RC Kuo PC Gupta GN. Plk-1 silencing in bladder cancer by sirna delivered with exosomes. Urology. (2016) 91:241.e1–7. doi: 10.1016/j.urology.2016.01.028 26876462

[57] Jin X Ma J Liang X Tang K Liu Y Yin X. Pre-instillation of tumor microparticles enhances intravesical chemotherapy of nonmuscle-invasive bladder cancer through a lysosomal pathway. Biomaterials. (2017) 113:93–104. doi: 10.1016/j.biomaterials.2016.10.036 27810645

[58] Yang D Zhang W Zhang H Zhang F Chen L Ma L. Progress, opportunity, and perspective on exosome isolation - efforts for efficient exosome-based theranostics. Theranostics. (2020) 10:3684–707. doi: 10.7150/thno.41580 PMC7069071 32206116

[59] Zhang Q Jeppesen DK Higginbotham JN Franklin JL Coffey RJ. Comprehensive isolation of extracellular vesicles and nanoparticles. Nat Protoc. (2023) 18:1462–87. doi: 10.1038/s41596-023-00811-0 PMC10445291 36914899

[60] Cao Y Feng J Zhang Q Deng C Yang C Li Y. Magnetic 3d macroporous mof oriented urinary exosome metabolomics for early diagnosis of bladder cancer. J Nanobiotechnol. (2024) 22:671. doi: 10.1186/s12951-024-02952-0 PMC11531116 39488699

[61] De Sousa KP Rossi I Abdullahi M Ramirez MI Stratton D Inal JM. Isolation and characterization of extracellular vesicles and future directions in diagnosis and therapy. Wiley Interdiscip Rev Nanomed Nanobiotechnol. (2023) 15:e1835. doi: 10.1002/wnan.1835 35898167 PMC10078256

[62] Erdbrrdbrn U Blijdorp CJ Bijnsdorp IV Borrsd FE Burger D Bussolati B. Urinary extracellular vesicles: A position paper by the urine task force of the international society for extracellular vesicles. J Extracell Vesicles. (2021) 10:e12093. doi: 10.1002/jev2.12093 34035881 PMC8138533

[63] Kural S Jain G Agarwal S Das P Kumar L. Urinary extracellular vesicles-encapsulated mirna signatures: A new paradigm for urinary bladder cancer diagnosis and classification. Urol Oncol. (2024) 42:179–90. doi: 10.1016/j.urolonc.2024.03.006 38594151

